# Activated Mesenchymal Stromal Cells Process and Present Antigens Regulating Adaptive Immunity

**DOI:** 10.3389/fimmu.2019.00694

**Published:** 2019-04-03

**Authors:** Kayleigh M. van Megen, Ernst-Jan T. van 't Wout, Julia Lages Motta, Bernice Dekker, Tatjana Nikolic, Bart O. Roep

**Affiliations:** ^1^Department of Diabetes Immunology, Diabetes and Metabolism Research Institute at the Beckman Research Institute of City of Hope, Duarte, CA, United States; ^2^Department of Immunohaematology and Blood Transfusion, Leiden University Medical Center, Leiden, Netherlands; ^3^Federal University of Minas Gerais (UFMG), Belo Horizonte, Brazil

**Keywords:** immune regulation, type 1 diabetes (T1D), antigen specific, immunotherapy, mesenchymal stromal cell (MSC), antigen presenting cell (APC)

## Abstract

Mesenchymal stromal cells (MSCs) are inherently immunomodulatory through production of inhibiting soluble factors and expression of immunosuppressive cell surface markers. We tested whether activated MSCs qualify for the induction of antigen-specific immune regulation. Bone marrow derived human MSCs were activated by interferon-γ and analyzed for antigen uptake and processing and immune regulatory features including phenotype, immunosuppressive capacity, and metabolic activity. To assess whether activated MSC can modulate adaptive immunity, MSCs were pulsed with islet auto-antigen (GAD65) peptide to stimulate GAD65-specific T-cells. We confirm that inflammatory activation of MSCs increased HLA class II, PD-L1, and intracellular IDO expression, whereas co-stimulatory molecules including CD86 remained absent. MSCs remained locked in their metabolic phenotype, as activation did not alter glycolytic function or mitochondrial respiration. MSCs were able to uptake and process protein. Activated HLA-DR3-expressing MSCs pulsed with GAD65 peptide inhibited proliferation of HLA-DR3-restricted GAD65-specific T-cells, while this HLA class II expression did not induce cellular alloreactivity. Conditioning of antigen-specific T-cells by activated and antigen-pulsed MSCs prevented T-cells to proliferate upon subsequent activation by dendritic cells, even after removal of the MSCs. In sum, activation of MSCs with inflammatory stimuli turns these cells into suppressive cells capable of mediating adaptive regulation of proinflammatory pathogenic T-cells.

## Introduction

Mesenchymal stromal cells (MSCs) are non-hematopoietic cells that can easily be sourced from various tissues, including bone marrow (1). They have been widely used clinically to improve the outcome of hematopoietic stem cell and solid organ transplants and to treat graft-vs. -host disease (2, 3). Consequently, safety has been established in terms of toxicity and tumerogenicity (3). The immunomodulative properties of MSCs also make these excellent candidates for cellular therapies targeting inflammatory and autoimmune disorders, including type 1 diabetes (T1D) (3). T1D is a T-cell mediated autoimmune disease in which autoreactive T-cells selectively kill insulin-producing beta-cells in the pancreas (4). Interestingly, MSCs have also been investigated for their potential to regenerate beta-cells, or to contribute to regeneration of beta-cells, which is another strategy to counter T1D (5, 6).

The hypoimmunogenic nature of MSCs could be responsible for evading alloreactivity as by definition they lack HLA class II (7). Hence, the use of allogeneic MSCs as a cellular therapy appears attractive as it is safe and enables “off-the-shelf” therapeutics (3). Immunomodulation by MSCs may be achieved by a range of soluble factors including indoleamine 2,3-dioxygenase (IDO) (1). In addition, cell-cell contact involving programmed death-ligand 1 (PD-L1) resulted in inhibition of T-cell proliferation and induction of T regulatory cells (2, 8).

Pro-inflammatory cytokines such as interferon gamma (IFN-γ) induce HLA class II expression on MSCs (9), which endorses antigen-presenting capacity of MSCs to CD4 T-cells, but could also affect their hypoimmunogenic nature. Indeed, mouse and human MSCs can act as unconventional antigen presenting cells, stimulating proliferation of T-cells (10–12, 14). Therefore, concerns have been raised about the potential to increase the immunogenicity of MSCs by activating them, but this has not consistently been substantiated (2). While cellular and humoral alloreactivity against MHC-mismatched MSCs have been reported in animal models, human MSCs did not show alloreactivity *in vitro* (13, 15). Indeed, activation of human MSCs enhanced their ability to inhibit allogeneic T-cell proliferation and reduced pro-inflammatory cytokine production in co-cultures (16–18).

Activation of MSCs may enable their use as an antigen-specific therapy, which is the long-sought objective in immunotherapy (19). While non-specific immunotherapies seem insufficient to intervene in auto-immune diseases and cancer (20), antigen-specific therapies using either antigenic peptide alone (21) or with cellular adjuvants such as antigen-pulsed dendritic cells (22, 23), or with CAR-T-cells (24), have emerged with promising outcomes. MSCs, too, have been tested as cell therapy to modulate adaptive immunity non-specifically (25–29). MSCs or their microvesicles inhibited an inflammatory response against diabetogenic peptides in patients with T1D and non-obese diabetic (NOD) mice (25, 26).

In the first clinical trial treating T1D patients, non-activated autologous MSCs preserved or even increased c-peptide response to a mixed meal tolerance test (MMTT) (30). This illustrates that their mere immunomodulatory nature may already affect the course of the disease favorably. Turning MSCs into antigen-specific adjuvants would increase the appeal to engage MSCs as a cellular therapy. This study set out to determine whether peptide-pulsed human MSCs can inhibit antigen-specific responses *in vitro* as a critical step to clinical translation of MSCs as an adaptive, antigen-specific immunotherapy in autoimmunity.

## Materials and Methods

### Human MSC Culture, Activation, and Antigen Processing

Bone marrow derived human MSCs were obtained from healthy individuals as described previously (31). Briefly, bone-marrow was collected from patients undergoing hip or knee replacement surgery at the Leiden University Medical Center (LUMC). Mononuclear cells were isolated by gradient centrifugation and cultured in “MSC medium” consisting of Dulbecco's Modified Eagle's (DMEM) low glucose medium (Life Technologies, New York, USA) with 10% Fetal Bovine Serum (FBS) (Sigma-Greiner, Wemmel, Belgium) and 100 IU/ml Penicillin and 100 IU/ml Streptomycin (Life Technologies). Next day, non-adherent cells were removed and cells were grown to confluence. Cells were harvested at ~90% confluency by trypsinizing the cells for 9 min at 37°C with 0.05% trypsin-EDTA (Life Technologies). The MSCs used for the current study have been characterized by flow cytometry and lineage differentiation in accordance with the minimal criteria for defining MSCs and used for clinical trials (32). In between passages cells could be cryopreserved in liquid nitrogen in 50% MSC medium, 40% FBS, and 10% Dimethyl Sulfoxide (DMSO). MSCs were collected and stored between passage 3 and 7.

Where applicable, MSCs were activated with 1,000 IU/ml IFN-γ (MSC-γ) (R&D systems) or by culturing MSCs in twice diluted supernatant of an autoimmune T-cell clone (PM1#11) isolated from a prediabetic patient and reactive to islet antigen glutamic acid decarboxylase 65 (GAD65) for 48 h (33). For antigen uptake and presentation, cells were incubated with labeled Ovalbumin (OVA-DQ, Invitrogen) that becomes fluorescent once it has been taken up and proteolytically degraded in the cell. 1 × 10^4^ MSCs were incubated with 5 μg OVA-DQ for 4 h at 37 or 4°C for control of spontaneous uptake/processing, and analyzed by flow cytometry and fluorescence microscopy (Xcyto-10). For microscopy, cells were visualized with Blue Mask (diluted 1:1,000 in PBS) upon 30 min incubation at room temperature.

### Human Monocyte Derived Dendritic Cells and T Cells

Monocyte-derived dendritic cells (DC) were generated as described previously (34). In short, peripheral blood mononuclear cells (PBMCs) were isolated from buffy coats of HLA typed healthy human donors (Sanquin, Amsterdam, The Netherlands) by density gradient centrifugation. Monocytes were selected by positive selection using CD14-specific magnetic beads (Miltenyi Biotec, Bergisch Gladbach, Germany) and cultured in RPMI-1640 (Life Technologies) supplemented with 8% fetal bovine serum (heat-inactivated FBS, Sigma F0804), 100 IU/mL Penicillin and 100 IU/mL Streptomycin (Pen/Strep, Life Technologies), 2 mM L-glutamin (Glut, Life Technologies), 500 IU/mL recombinant IL-4 (Invitrogen, Breda, Netherlands) and 800 IU/mL recombinant GM-CSF (Invitrogen) for 6 days to obtain immature DC (iDC). iDC were matured in a 2-day culture using 100 ng/mL lipopolysaccharide (LPS: Sigma-Aldrich Chemie, Zwijndrecht, The Netherlands). Dendritic cells used in all experiments were HLA-matched to the PM1#11 clone (HLA-DR3). CD14 negative cells were preserved in liquid nitrogen and used in different assays as Peripheral Blood Lymphocytes (PBLs). The PM1#11 clone was derived from a prediabetic patient after informed consent. The clone is HLA-DR3 restricted and specific for GAD65_339−352_ (33). Cells were cultured in Iscove's Modified Dulbecco's Medium (IMDM; Lonza) supplemented with 10% pooled human serum, Pen/Strep, and glutamine (Glut).

### Cytokine Assays

Supernatants from activated and non-activated MSCs and GAD-specific T-cell clones were harvested and analyzed for cytokine analysis with a Luminex kit (Bio-Rad; Hercules, CA) according to the manufacturer's protocol.

### Flow Cytometry

MSCs were stained with 1:5,000 Live/Dead Fixable Blue Dead Cell Stain Kit (Life Technologies) for 20 min according to manufacturer's protocol, after which cells were incubated with a panel of monoclonal antibodies (Supplemental Table S1) for 30 min on ice. Cells were washed in FACS buffer containing 1% FBS and 0.05% Sodium Azide (Sigma-Aldrich) and analyzed using FACS Canto and Fortessa (BD). Data was analyzed using FACS DIVA v8 (BD Biosciences) and FlowJo v10 software (Ashland, Oregon, USA). The gating strategy is presented in the supplement (Supplemental Figure S1).

### Real-Time Metabolic Characterization

The XF^e^96 extracellular flux analyzer (Seahorse Bioscience, North Billerica, USA) was used to measure mitochondrial oxygen consumption rate (OCR, O_2_ mpH/min) and extracellular acidification rate (ECAR, mpH/min). Prior to experiments, optimization with regards to cell number and concentration of compounds was performed. Subsequently, MSCs were harvested, counted, and plated (1 × 10^4^ cells/well) in MSC medium supplemented or not with 1,000 IU/mL IFN-γ and incubated for 48 h at 37°C. On the day of analysis, MSCs were thoroughly washed (3x) in either glycolysis stress test assay medium (DMEM base, 2 mM L-glutamine; pH 7.35) or mitochondrial stress test medium (DMEM base, 2 mM L-glutamine, 1 mM pyruvate, 25 mM glucose; pH 7.35) and incubated in a non-CO_2_ incubator at 37°C for 1 h. For the glycolysis stress test the following compounds were used in the subsequent stages: basal (no drugs), glycolysis (10 mM glucose), glycolytic capacity (1 μM oligomycin), and glycolysis inhibition (50 mM 2-DG). For the mitochondrial stress test the following compounds were used in the subsequent stages: basal respiration (no drugs), ATP production inhibition (1 μM oligomycin), maximal respiration (0.5 μM FCCP), and electron transport chain inhibition (0.5 μM rotenone and 0.5 μM antimycin A).

### Alloresponse, Suppression, and Antigen-Specific Proliferation Assays

HLA-typed human PBLs or PM1#11 cells were labeled with CellTrace™ carboxyfluorescein succinimidyl ester (CFSE) (Invitrogen) by staining 1 × 10^6^ cells/mL in PBS with 0.5 μg/mL CFSE for 2 min at room temperature. Subsequently, 1 × 10^5^ CFSE-labeled cells were plated in a 96-well plate and used for the following assays. For all assays, cells were harvested after 4 days of culture and stained with monoclonal antibodies against CD3, CD4, and CD8 and analyzed for proliferation by flow cytometry, unless otherwise described.

PBL cells were incubated with either HLA-mismatched MSCs, MSC-γ, or DC at a ratio 10:1 or CD3/CD28 beads (ratio 1:1) to measure alloresponse (Dynabeads Human T-Activator, Thermo Fisher). PBL cells were stimulated with CD3/CD28 beads alone (ratio 1:1) or in the presence of MSC or MSC-γ (ratio PBL:MSC 10:1) to test suppressive capacity of MSCs. MSC-γ or DCs prepulsed for 4 h with 5 ug/mL GAD65_339−352_ peptide or GAD65 protein, after being washed three times, were incubated with GAD-specific T-cells (PM1#11) to assess antigen-specific proliferation.

HLA-DR3 (matched) and HLA- DR13 (mismatched) MSC-γ were prepulsed with different concentrations of the GAD65_339−352_ peptide (0.2, 1, 5 μg/mL) for 4 h and thoroughly washed for the antigen-specific inhibition co-culture experiment. Next, HLA-DR3 DCs (HLA-matched), prepulsed with 1 μg GAD65_339−352_ peptide, and GAD-specific T-cells were added to the culture in a DC:MSC:PM1#11 ratio of 1:1:5. After 3 days, [^3^H]-thymidine (0.5 μCi/well) was added for 18 h, after which incorporation was measured using a liquid scintillation counter. Data shown is the mean of triplicates with the standard error of the mean (SEM). This experiment was replicated with CFSE-labeled PM1#11 cells.

HLA-DR3 matched or -mismatched MSC-γ were loaded with GAD peptide and incubated with GAD-specific T-cells in a ratio 1:10 MSC:T cell for the preconditioning assay. After 24 h, T-cells were harvested leaving adherent MSCs intact, washed in PBS and subsequently the T-cells were cultured with DCs prepulsed with 1 μg/mL GAD peptide (ratio 1:10 DC:T cell). A proliferation index (average number of divisions by dividing cells) was calculated by dividing the total number of divisions by the number of T-cells that proliferated.

### Statistical Analysis

Data were analyzed for statistical significance using unpaired Student's *t*-test, one-way ANOVA or two-way ANOVA with, where appropriate, subsequent Tukey or Sidak's post-test for multiple comparisons using GraphPad Prism 7 (GraphPad Software, La Jolla, USA). In the figure legends is described which test is used. A *p* < 0.05 was considered significant.

## Results

### Activation of MSCs Increases HLA-DR Expression and Immune Inhibitory Markers, While Maintaining Their Metabolic Profile

**Figure 1:**
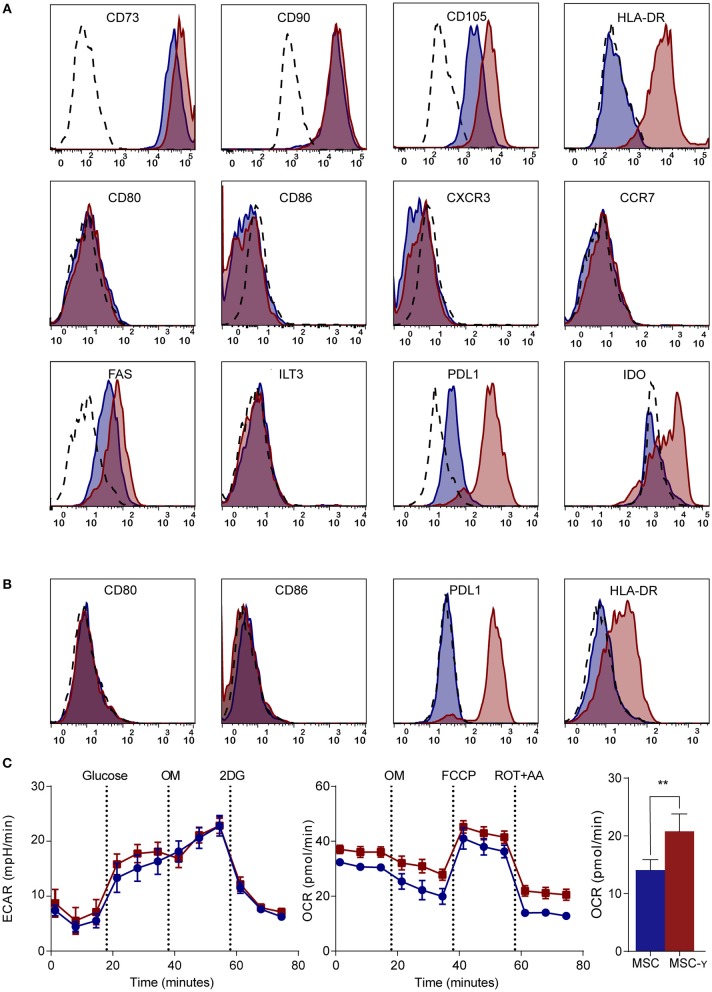
Phenotype and metabolism of activated MSCs. **(A,B)** Flow cytometric analysis of the phenotype of non-activated (blue histograms) and activated (red histograms) MSCs and isotype controls (dashed histograms). In **(A)** activation is by IFN-γ and in **(B)** by supernatant of non-activated (blue) or activated (red) GAD-specific T-cells. The first row shows markers that identify MSCs; the second row represents co-stimulatory molecules and chemokine receptors; the third row identifies inhibitory markers. Representative histograms are shown (*N* = 4). **(C)** Representative graphs of real-time metabolic data of non-activated (blue) and activated (red) MSCs as analyzed by the XF^e^ extracellular flux analyzer (Seahorse). In the glycolysis stress test (left graph) glucose is injected which induces glycolysis. Next, oligomycin (OM) is injected to induce maximal glycolytic capacity and 2-deoxy-D-glucose (2-DG) finally to inhibit glycolysis. In the mitochondrial stress test (right graph), basal respiration is measured, after which OM is added to inhibit ATP production. Consequently, carbonyl cyanide-4-phenylhydrazone (FCCP) is injected to induce maximal respiratory capacity. Lastly, rotenone and antimycin-A is added to block the electron transport chain. Only the non-mitochondrial respiration (bar graph) was significantly increased in activated MSCs (MSC-γ) compared to non-activated MSCs (MSC) (*N* = 4). An unpaired student's *t*-test was used to test statistical significance. ^**^p = 0.006. ECAR, Extracellular Acidification Rate; OCR, Oxygen Consumption Rate.

MSCs generally lack HLA-DR expression, while this is needed for antigen presentation to CD4 T-cells (7). Activation of MSCs by IFN-γ increased the expression of HLA-DR without decreasing the expression of markers that characterize MSCs (CD73, CD90, and CD105) (Figure 1A). Markers typically lacking on resting MSCs, namely CD34, CD45, CD14, and CD19 remained negative after activation (32) (Supplemental Figure S2). Besides these standard markers to characterize MSCs, activated MSCs were phenotyped more extensively by flow cytometry, analyzing expression of activating and inhibiting molecules involved in antigen-presentation and T-cell stimulation (35). Activation of MSCs with IFNg did not increase CD86 or CD80 expression. Similarly, chemokine receptors CCR7 and CXCR3, which are implicated in migration to lymph nodes and inflamed tissues (36), respectively, were not expressed before or after activation. Yet, activation of MSCs did enhance the expression of inhibitory molecule PD-L1, death receptor FAS and intracellular IDO expression, whereas inhibitory molecule ILT3 showed no change (Figure 1A). Next, we stimulated MSCs with the supernatant of activated autoreactive Th1-cells that we deem a more (patho)physiologically relevant stimulation when mimicking inflammatory insulitis than a single cytokine stimulation. The supernatant of the activated Th1-cells contained substantially higher levels of pro-inflammatory cytokines (IL-1b, IL-2, IL-5, IL-6, IL-8, IL-12, IL-13, IL-17, IFN-γ, and TNF-α), compared to the non-activated Th1-cell supernatant (Supplemental Figure S3). Similar to activation with IFN-γ alone, activating MSCs with activated Th1-cell supernatant increased expression of HLA-DR and PD-L1 while keeping CD80 and CD86 expression low (Figure 1B). The supernatant of non-activated autoreactive Th1 did not activate MSCs in terms of surface marker expression. In addition to surface marker expression, we analyzed cytokine secretion by activated MSCs. Even after activation the secretion of pro- and anti-inflammatory cytokines (IL-2, IL-4, IL-5, IL-10, IL-12, IL-13, TNF-α) was low and was not increased compared to non-activated MSCs (Supplemental Figure S4).

Since the metabolism of immune cells has proven pivotal in directing their immune activation or quiescence (37), a real-time metabolic characterization was performed to assess the effect of MSC activation on their metabolism. Activation did not impair the metabolism of MSCs, as indicated by unchanged mitochondrial respiration and glycolysis using a Seahorse analysis (Figure 1C). Yet, non-mitochondrial respiration (measured by OCR) was increased in MSCs upon activation (*p* = 0.006; unpaired student's *t*-test) (Figure 1C).

In summary, activation of MSCs enables their interaction with CD4 T-cells by upregulating HLA class II and selectively reinforces their inhibitory properties through increasing PD-L1 expression but not co-stimulatory molecules CD80 and CD86, while maintaining their resting metabolic profile.

### Activated MSCs Do Not Stimulate Allo-Reactive T Cells but Enhance Immunosuppressive Capacity

**Figure 2:**
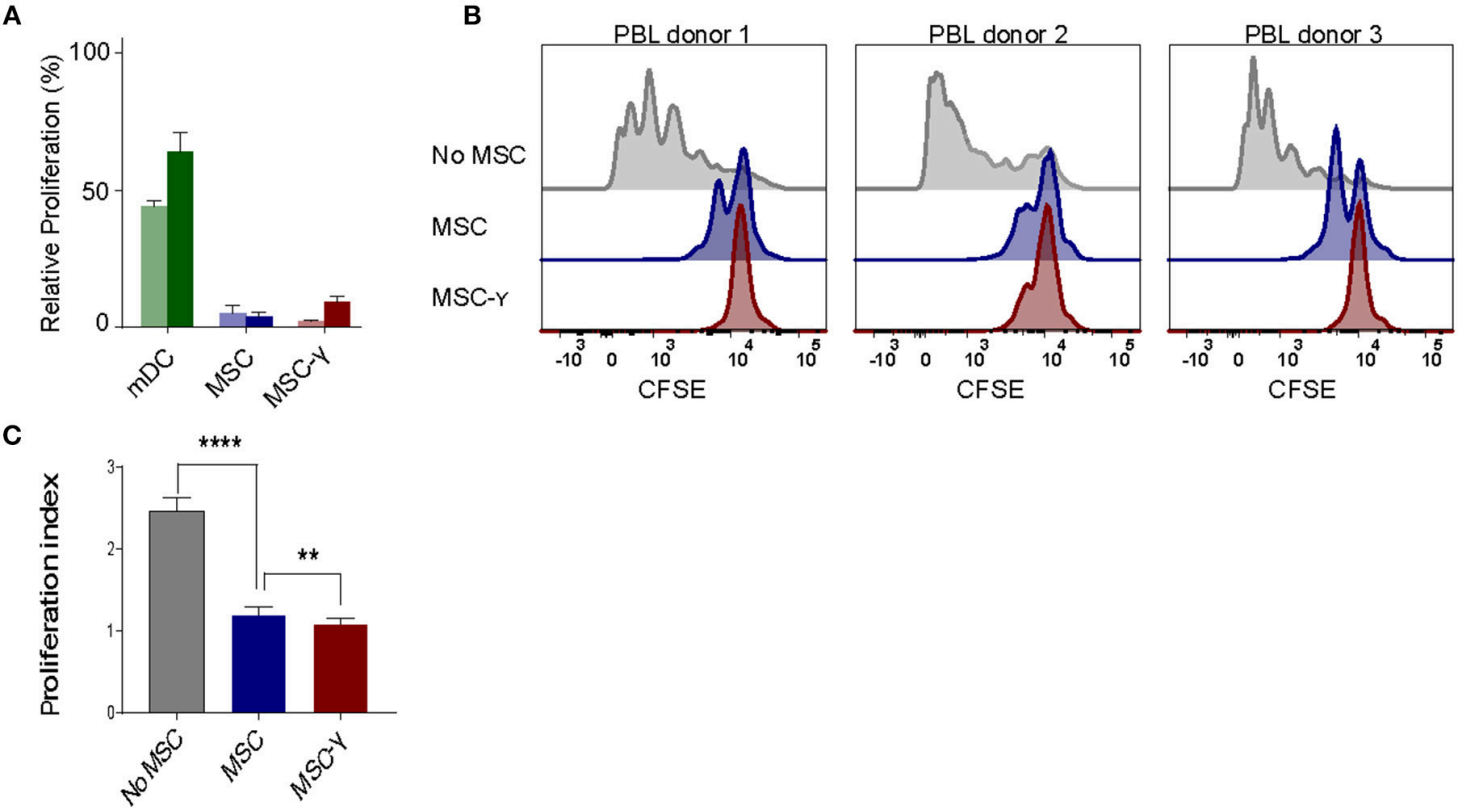
Allo-response and immunosuppressive capacity of activated MSCs. **(A)** A mixed lymphocyte reaction (MLR) was performed. Proliferation of CFSE-labeled PBLs from two independent donors (dark and light bar) in a co-culture with HLA-mismatched MSCs (blue bars), or MSC-γ (red bars). HLA-mismatched mDCs (green bars) were used as a positive control of proliferation of allo-reactive T-cells. Proliferation was calculated relative to CD3/CD28 bead induced proliferation of PBLs (set to 100%) (*N* = 2). **(B)** A suppression assay was performed with three independent PBL donors. Proliferation of CFSE-labeled PBLs was induced by CD3/CD28 beads. Histograms represent proliferation of CD4 T-cells when stimulated with CD3/CD28 beads alone (gray histograms), or in the presence of non-activated MSC (blue histograms), or MSC-γ (red histograms). The panels represent proliferation of three different allo-geneic PBL donors in co-culture with one MSC donor. **(C)** This experiment was repeated three times, each time with different PBL and MSC donors. The bar graph shows the proliferation index of different allogeneic CFSE-labeled PBL donors, activated by CD3/CD28 beads, cultured with no MSC (gray), MSC (blue), and MSC-γ (red). The proliferation index is on gated CD4 T-cells. The data are presented as mean ± SD of three different MSC donors each cocultured with different allogeneic PBL donors in three independent experiments. ^**^p = 0.008; ^****^p < 0.0001, one-way ANOVA with Tukey's correction for multiple comparisons. PBL, peripheral blood lymphocyte.

The lack of HLA-DR expression promotes the immune privileged state of MSCs (1). This would imply that inducing HLA-DR expression on activated MSCs may cause concerns regarding increasing exposure to allo-reactive CD4 T-cells. To investigate this, non-activated or IFNγ-activated MSCs (MSC-γ) were cocultured with HLA-mismatched lymphocytes (PBL) and T-cell proliferation was measured using a CFSE-dilution assay. No proliferation of HLA-mismatched CD4+ T cells was observed in response to MSC or MSC-γ, whereas dendritic cells with the same HLA class II mismatch as the MSCs did stimulate proliferation of CD4 T-cells (Figure 2A). Next, the immunosuppressive potential of MSCs and MSC-γ was assessed in a co-culture of MSC or MSC-γ with HLA-mismatched PBLs that were activated with CD3/CD28 beads. CD3/CD28 beads induced T-cell proliferation that was inhibited by MSC-γ and to a lesser extent by non-activated MSCs (Figure 2B). Non-activated MSCs significantly inhibited CD3/CD28 bead-stimulated proliferation of HLA-mismatched CD4 T-cells (*p* < 0.0001) and activation of MSCs significantly enhanced this inhibitory potential, compared to non-activated MSCs (*p* = 0.008; one-way ANOVA with Tukey's correction for multiple comparisons) (Figure 2C).

### MSCs Take Up and Process Antigen, but Do Not Induce T-Cell Proliferation

**Figure 3:**
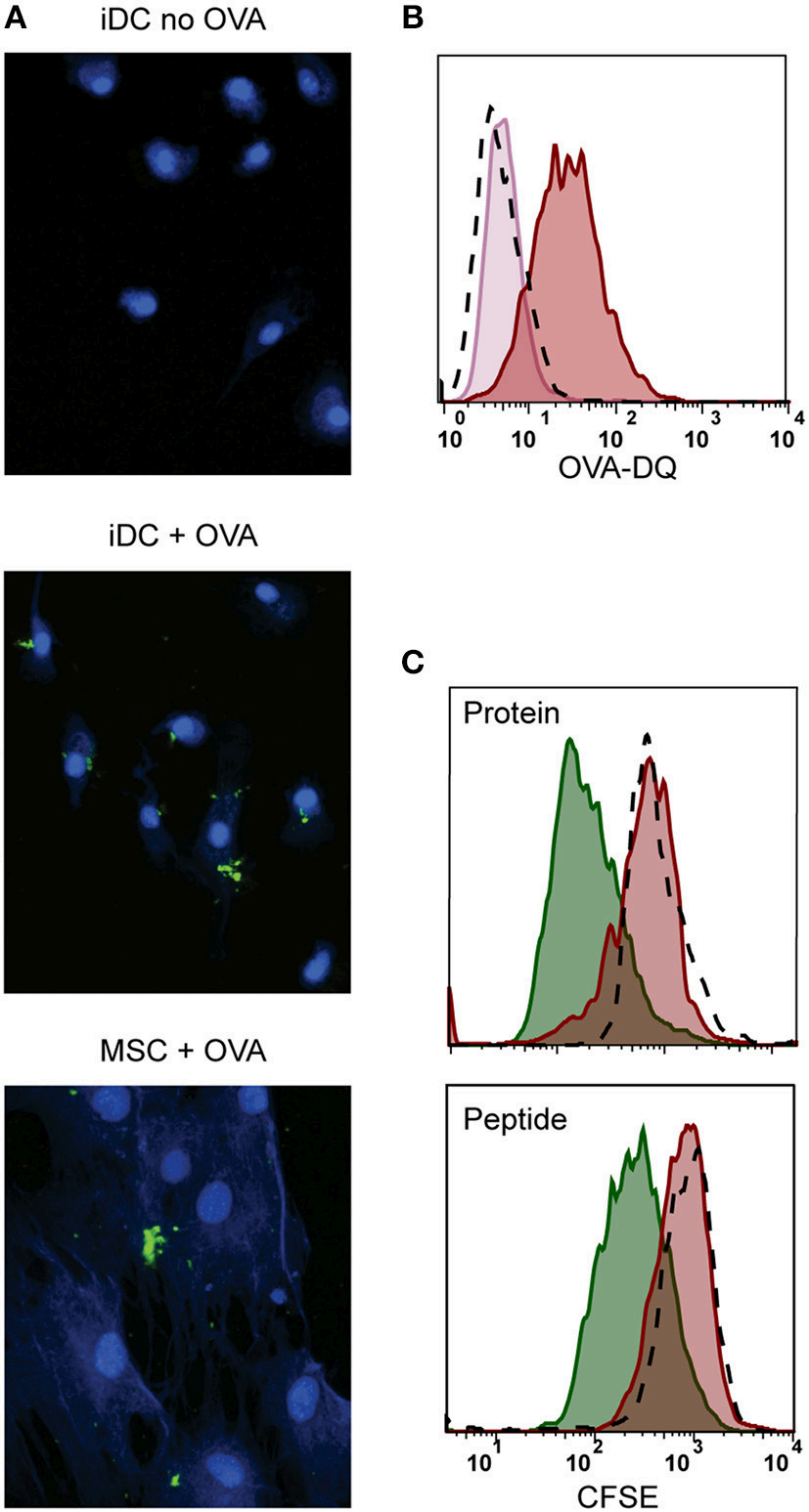
Antigen processing and induction of T-cell proliferation by activated MSCs. Firstly, the antigen uptake and processing capacity of MSCs was tested. MSCs were pulsed with OVA-DQ that only fluoresces once it has been taken up and proteolytically degraded in the cell. **(A)** Fluorescence microscopy pictures of processed OVA-DQ (green) by MSCs or immature dendritic cells (iDC). Blue Mask shows nuclei and cytoplasm defining individual cells. **(B)** Histograms depict the fluorescence of processed OVA-DQ in MSCs measured by flow cytometry after 4 h incubation at 4°C (pink histogram) or 37° (red histogram) and isotype control (dashed histogram). **(C)** Consequently, the capacity of MSCs to induce proliferation of an antigen-specific T-cell was tested in a co-culture. MSCs or DCs were pulsed with GAD protein or GAD peptide and were both HLA-matched to the T-cell clone. Proliferation of T-cells was measured with CFSE dilution after 4 days of co-culture. The histograms present proliferation of CFSE-labeled GAD-specific T-cell clone upon activation with GAD-pulsed DCs (green histograms), GAD-pulsed MSC-γ (red histogram), or unpulsed MSC-γ (dashed histogram). All histograms are representative of 4 independent experiments.

We further explored whether the immunosuppressive properties of MSCs could be combined with antigen presentation. For that, besides HLA class II expression, antigen-presenting cells need to take up and process antigen (38). We tested the antigen uptake and processing capacity of MSCs by incubating with fluorescent quenched Ovalbumin protein (OVA-DQ) that only emits light once it has been taken up and proteolytically degraded in the cell. MSCs were able to take up and process OVA-DQ, as demonstrated by the detection of a fluorescent signal by both microscopy (Figure 3A) and flow cytometry (Figure 3B). Next, to test whether the uptake and processing of an antigen by MSCs could induce antigen-specific T-cell proliferation, activated MSCs expressing HLA-DR3 were pulsed with either whole protein (GAD65) or peptide (GAD65_339−352_) and cocultured with HLA-DR3-restricted GAD65_339−352_-specific T-cells. Neither whole protein nor peptide prepulsed HLA-matched MSC-γ induced proliferation of GAD65_339−352_ specific effector T-cell clones, whereas prepulsed, HLA-matched DCs did (Figure 3C).

### MSCs Impede Proliferation of Activated Antigen-Specific T-Cells, Imprinting the Inhibition Even After Their Removal

**Figure 4:**
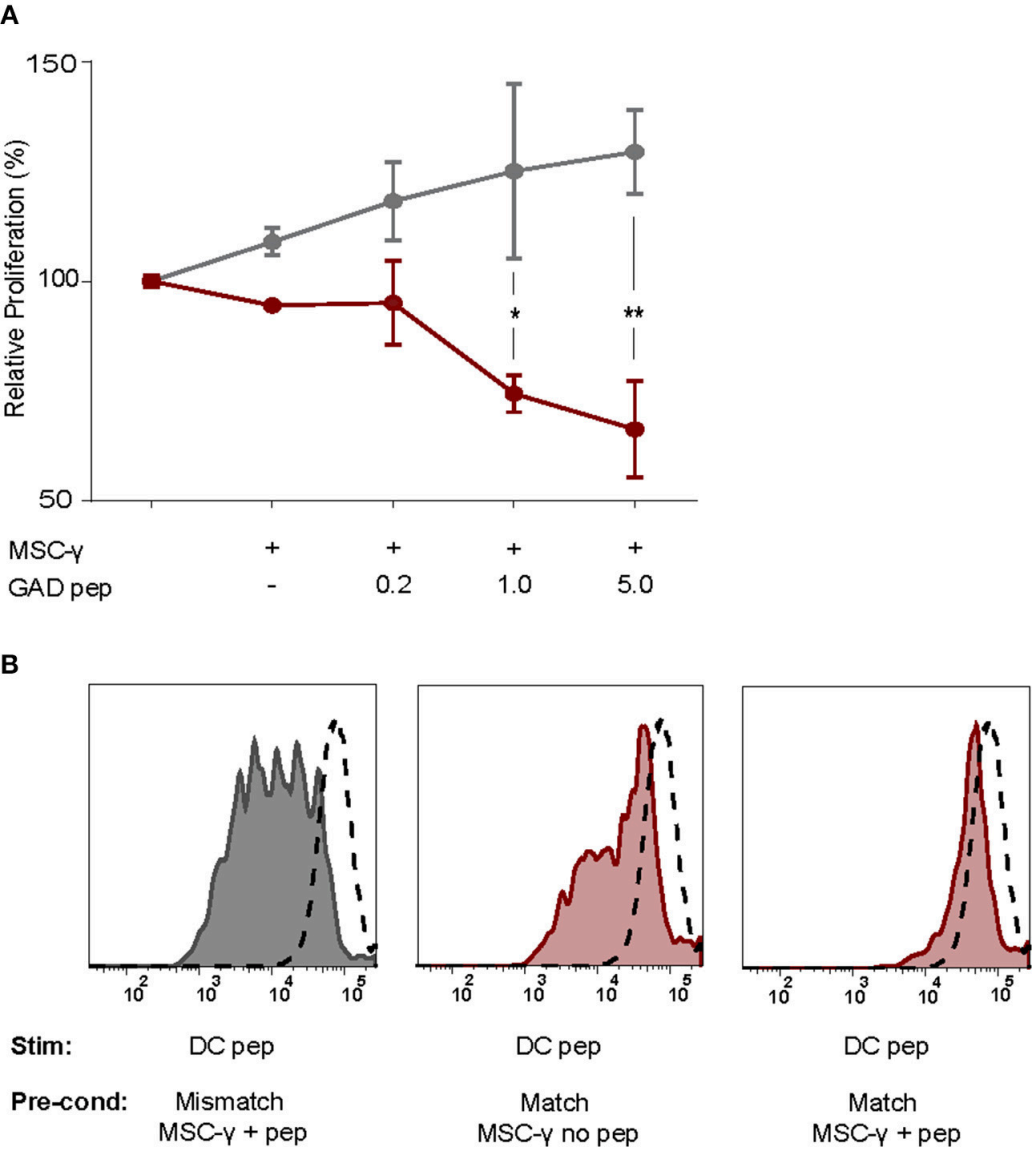
Antigen-specific inhibition of proliferation by activated MSCs. The capacity of MSCs to inhibit antigen-specific proliferation of a T-cell clone was assessed. **(A)** Proliferation of a GAD-specific T-cell clone was induced by HLA-matched and peptide-pulsed DCs, which was set to 100%. HLA-mismatched MSC-γ (gray symbols) or HLA-matched MSC-γ (red symbols) that were prepulsed with increasing concentrations of GAD peptide (GAD pep) were added to the DC and T-cell co-culture. Prepulsing HLA-matched MSCs with GAD peptide significantly inhibited proliferation of a GAD-specific T-cell clone, compared to HLA-mismatched MSCs: 1 μg/mL (^*^p = 0.013) and 5 μg/mL (^**^p = 0.003). Difference was tested using a two-way ANOVA with Sidak's correction for multiple comparisons. **(B)** Conditioning experiment to test whether MSCs are needed in the co-culture to inhibit antigen-specific T-cell proliferation. GAD-specific T-cell clones were preconditioned (Pre-cond) for 24 h by HLA-mismatched (gray histogram) MSC-γ, or HLA-matched (red histogram) MSC-γ pulsed (right panel) or not pulsed (middle panel) with GAD peptide (pep). Consequently, the GAD-specific T-cell clone was harvested and stimulated (Stim) with HLA-matched and peptide-pulsed DCs. Proliferation of T-cells was measured with CFSE dilution. Proliferation of unstimulated T-cells is depicted in the dashed histogram. All histograms and graphs are representative of two independent experiments.

As activated MSCs pulsed with antigen did not induce T cell proliferation, we tested whether they modulate islet autoreactive T-cells by actively inhibiting T-cell proliferation instead. Proliferation of GAD-specific T-cells was induced by DCs expressing HLA-DR3 and GAD peptide. T-cell proliferation was assessed in the presence of DCs alone or together with activated MSCs pulsed with different concentrations of GAD peptide prior to the co-culture. The T-cell proliferation was indeed induced by DC alone and did not change in the presence of GAD-peptide pulsed, activated MSCs carrying HLA-DR13 that is irrelevant for these T-cells. If anything, the proliferation of T cells in the presence of pulsed MSCs with mismatched HLA tended to increase although not significantly. The stimulation of GAD-specific T cells in the presence of HLA-DR3 MSCs was reduced in a peptide-dose dependent manner, compared to HLA-DR13 MSCs (GAD peptide 1 μg/mL *p* = 0.013; 5 μg/mL *p* = 0.003; Figure 4A), underscoring the need for HLA-matching to induce antigen-specific T-cell inhibition by activated antigen-pulsed MSCs.

To test whether the presence of MSCs in the co-culture is necessary to inhibit the DC-induced proliferation of auto-reactive T-cells, GAD-specific T-cells were preconditioned for 24 h with MSCs that were either HLA-matched (DR3) or mismatched (DR13) with or without peptide. Next, the non-adherent T-cells were harvested carefully from adherent MSCs, washed and transferred to new co-cultures with peptide-pulsed HLA-matched (DR3) DCs. T-cell proliferation was measured by CFSE dilution after 4 days of co-culture with DCs (Figure 4B). When preconditioning was performed with activated and HLA-matched MSCs pulsed with peptide, proliferation of T-cells was totally abolished in the subsequent DC co-culture. In contrast, T cells proliferated when pre-conditioned with activated and peptide-pulsed DR13 MSC. Also, T cell proliferation was only marginally inhibited if the preconditioning of matched and activated MSC occurred in the absence of antigen.

In summary, only conditioning of antigen-specific T-cells by activated and HLA-matched MSCs pulsed with antigen inhibited subsequent DC-stimulated T-cell proliferation, even upon removal of the MSCs.

## Discussion

MSCs have shown great promise as an immune-modulating therapy in the clinic for several diseases, but thus far they have been solely explored an antigen-non-specific therapy (3). Combining the immunosuppressive properties of MSCs with antigen-presenting qualities would create an attractive cellular product for immune modulation. In this study, we provide *in vitro* evidence that activated MSCs can take up and process antigens and upregulate HLA class II expression, collectively granting MSCs the conditions necessary to transform into unconventional antigen-presenting cells.

We confirmed that MSC activation does not alter their hypoimmunogenic profile. Although HLA-DR expression was increased after activation, this was not accompanied by an increase in activating co-stimulatory molecules CD86 and CD80. Activation of MSCs did also not change expression of chemokine receptors CCR7 and CXCR3, implicated in migration to lymph nodes and inflamed tissues, respectively (36). Instead, activation increased the expression of immunosuppressive checkpoints such as PD-L1 and IDO, both known to endorse MSCs with immuno-modulatory capacities (39, 40). While high concentrations of IFN-γ alone activated MSCs, we now show that the cytokines secreted by antigen-stimulated T-cells could activate MSCs in a similar fashion, suggesting that inflammation *in vivo* may reinforce immunosuppressive capacity of MSCs without increasing their immunogenicity. We propose that this extended Th1 cytokine profile is more representative of an actual T-cell response to antigen than the rather excessive and selective cytokine(s) usually tested to mimic inflammation. This increase in inhibitory markers matches our findings that activation of MSCs actually reinforces their immunosuppressive capacity in a mixed lymphocyte reaction.

Non-activated MSCs are highly glycolytic (41), which has been linked to their immunosuppressive capacity (42), while mitochondrial respiration proved less important to the suppressive functionality of MSCs (41). Our findings point toward a stable metabolic phenotype in terms of mitochondrial respiration and glycolysis after activation of MSCs. Yet, non-mitochondrial respiration was increased upon activation, which could signify a more extensive usage of desaturases and detoxification enzymes (43).

Taking up and presenting antigens on HLA class II molecules is a *sine qua non* of antigen-presenting cells (38). Activated MSCs in our study were able to take up and process antigen in line with previous findings (10–12). While activation and peptide-pulsing of MSCs did not induce proliferation of peptide-specific T-cells, they were able to inhibit the proliferation of autoreactive T-cells in an antigen-specific manner. Peptide-pulsing alone did not transform MSCs into suppressive cells, as activated and peptide-pulsed but HLA-mismatched MSCs did not inhibit T-cell proliferation. We show that MSCs interfere in T cell activation induced by professional APC (i.e., DCs), as well as endorse an inhibitory effect in T cells lasting beyond their presence. Indeed, it is intriguing that a 24-h preconditioning of T-cells with MSC loaded with their antigen was sufficient to change the course of events of those T-cells in the subsequent 4 days after removal of MSCs. It should be noted that the T-cells were washed after the MSC preconditioning so the effect on T-cell inhibition cannot be accounted for by soluble factors or microvesicles of MSCs.

Our finding that HLA class II matching with the recipient is required in order to deliver adaptive immune alterations implies that the suppressive licensing by MSCs is a direct consequence of peptide presentation on the appropriate HLA restriction elements to the T-cell. This is the case for both intervening in an antigen-specific response as for preventing the induction of a response. In fact, proliferation of GAD-specific T-cells was slightly increased in case the MSC had a different HLA than the T-cell. We propose that this is due to increased presentation by dendritic cells of peptides that had leaked from peptide-pulsed MSCs during co-culture, as increasing concentrations of peptide used to pulse MSCs resulted in a slight increase in T-cell proliferation. Similar leak or “delivery” of antigen by MSCs to DCs has been reported (12). Collectively, antigen-specific immune modulation by activated MSCs was dependent upon the presence of the relevant islet peptide epitope, the appropriate HLA-DR3 restriction element for presentation of the islet epitope, and showed an epitope dose-dependent increase in inhibition of T-cell proliferation. Nevertheless, matching MSCs for one HLA-haplotype with the T-cell donor was sufficient to inhibit antigen-specifically. This increases the number of potential MSC recipients in an off-the-shelf therapy, while limiting the risk of alloreactivity (13). No allo-response was provoked by MSCs *in vitro* in our studies, even when these were induced to express completely mismatched HLA class II.

In terms of underlying mechanism, our data suggests that the antigen-specific inhibition of T-cell proliferation by MSCs results from antigen presentation in HLA class II in the absence of co-stimulatory activation, similar to tolerogenic dendritic cells (34), as activated MSCs lack CD80 and CD86. Cytokine mediated modulation seems unlikely, since activation of MSCs did not affect their cytokine secretion profile. We favor the possibility of a role for the inhibitory molecules PD-L1 and IDO, which are both increased on activated MSCs and could lead to an inhibitory rather than stimulatory signal to T-cells. In concert, this may result in suppression of an adaptive (auto)immune response.

Inconsistencies have been reported with regard to adaptive features of MSCs upon activation between mice and men, and between human studies. One discrepancy was noted between mice and men that relates to MSC density, which inversely affected their antigen processing and MHC class II upregulation (44). We also found that activation and peptide-pulsing of human MSCs resulted in inhibition of T-cells, whereas Ag-pulsed and activated mouse MSCs activated T-cells (11). Their T-cell activation was CD80 dependent, whereas human MSCs do not express CD80 upon activation, which we confirm (45). In terms of inconsistencies between human studies, one report claimed that antigen presenting and stimulating qualities of MSCs were uniquely induced by low levels of IFN-γ, whereas HLA class II was decreased at higher IFN-γ levels, while we show that MSCs also express HLA class II at high IFN-γ exposure (10). Yet, our data are consistent with their observation that immune suppression would be more pronounced during severe inflammation. Furthermore, these MSCs were shown to induce antigen-specific T-cell proliferation in a short co-culture (10), while we found immune suppression. This discrepancy might be explained by duration of co-culture (18). Indeed, human MSCs were reported to inhibit T-cell activation in long-term cultures, which we confirm, whereas shorter cultures activated T-cells. To conclude, our *in vitro* data are consistent with other human studies with similar conditions and point to an antigen-specific suppressive role for MSCs, which further supports the hypoimmunogenic profile of MSCs (1, 46).

We here provide proof-of-concept that activated MSCs could take up and process antigen and inhibit proliferation of activated effector T-cells in an antigen-specific manner, without overtly increasing the immunogenicity of allogeneic MSCs. These features provide encouraging first steps in the clinical translation of the use of pre-activated MSCs as a cellular immune intervention therapy. This could pave the way to use activated HLA-haplotype matched allogeneic MSCs as immunomodulatory therapeutic cell products for intervention in adaptive immunity in autoimmune disease.

## Ethics Statement

This study was carried out in accordance with the recommendations of the Medical Ethics Board of the LUMC with written informed consent from all subjects. All subjects gave written informed consent in accordance with the Declaration of Helsinki. The protocol was approved by the Medical Ethics Board of the LUMC.

## Author Contributions

KvM and E-JvW conducted experiments, analyzed the data, and wrote the manuscript. JL and BD conducted experiments and edited the manuscript. TN designed and conducted experiments, analyzed the data, and revised the manuscript. BR conceived the idea, designed experiments, analyzed data, revised the manuscript, and contributed to the discussion.

### Conflict of Interest Statement

The authors declare that the research was conducted in the absence of any commercial or financial relationships that could be construed as a potential conflict of interest.
