# Supplementary material for: Activated Mesenchymal Stromal Cells Process and Present Antigens Regulating Adaptive Immunity
Source: Front Immunol. 2019 Apr 3;10:694. doi: 10.3389/fimmu.2019.00694 (PMC6457321; doi:10.3389/fimmu.2019.00694)
Supplement: Supplementary file 1 [file Data_Sheet_1.pdf]

Supplemental table S1. List of anti-human monoclonal antibodies used for MSC phenotyping

Target	Clone	Fluorochrome	Company
CD73	AD2	PE-Cy7	Biolegend
CD90	Thy-1A1	APC	R&D systems
CD105	43A3	PE	Biologend
HLA DR	G46-6	BV605	BD Biosciences
CD80	BB1	FITC	BD Pharmigen
CD86	L307.4	PE	BD Pharmigen
CXCR3	1C6	PE	BD Pharmigen
CCR7	3D12	FITC	eBiosciences
FAS	DX2	FITC	Biolegend
ILT3	ZM3.8	PE-Cy7	Beckman Coulter
PDL1	MIH1	PDL-1	eBiosciences
IDO	Eyedio	PE	eBiosciences
CD3	UCHT1	AF700	BD Biosciences
CD4	RPA-T4	APC-Cy7	BD Biosciences
CD14	61D3	FITC	Thermo Fisher
CD19	HIB19	PerCP-Cy5.5	Biolegend
CD34	561	AF700	Biolegend
CD45	H130	BV421	BD Biosciences

Supplemental figure S1. (A) Gating strategy MSCs. (B) Gating strategy lymphocytes.

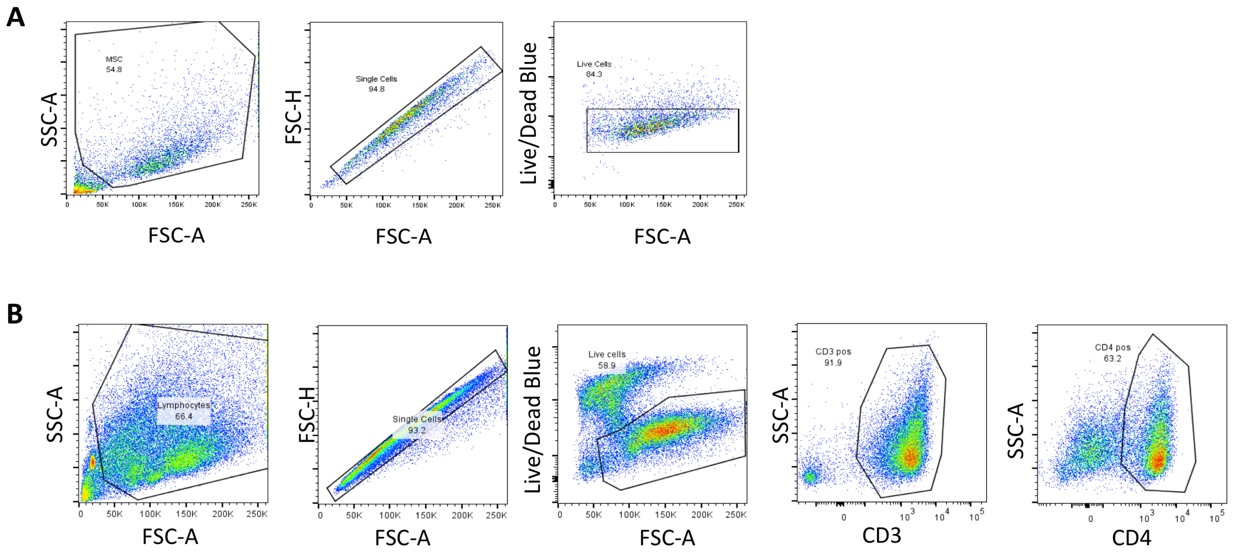

Supplemental figure S2. Negative markers of MSCs.

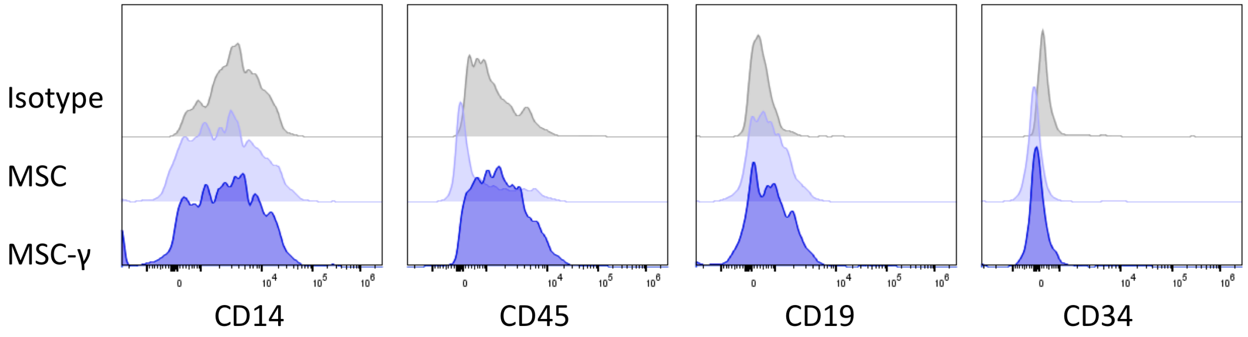

Representative histograms of negative markers for non-activated (MSC) and activated MSCs (MSC-γ), compared to isotype controls. N= 5.

Supplemental figure S3. Cytokine production of activated and non-activated GAD-specific T-cell clone.

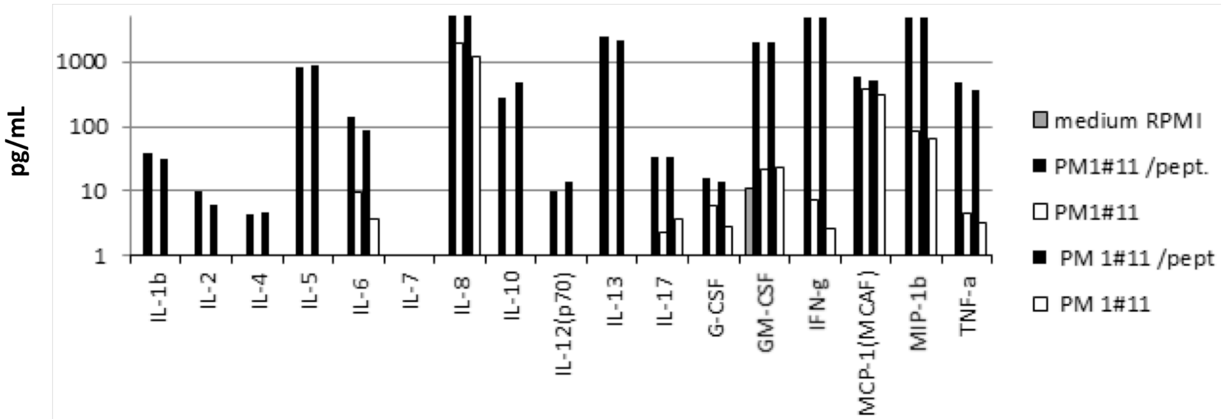

Cytokine profile of GAD65 T-cell clone upon GAD65 peptide stimulation. Results are shown as result of two independent experiments. Grey bars show the cytokine concentration in media (RPMI), white bars the cytokine profile of resting GAD65 T-cells and the black bars indicate the peptide specific cytokines release.

Supplemental figure S4. Cytokine production of activated and non-activated MSCs.

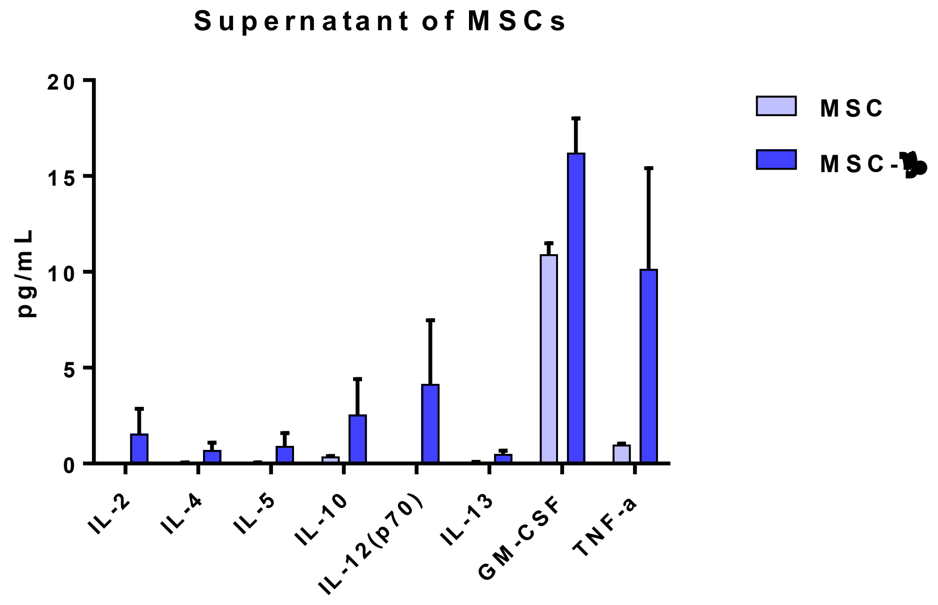

Luminex assay of the supernatant of non-activated (MSC) and activated MSCs (MSC-γ). The data are presented as mean ± SD of three different MSC donors.
